# 1678. Non-Invasive and invasive Pediatric *Group A Streptococcus* Infections (iGAS) Admissions During 2021 and 2022 at a Tertiary Health Care Center: Resurgence and Concomitant Viral Infections

**DOI:** 10.1093/ofid/ofad500.1511

**Published:** 2023-11-27

**Authors:** Susan Fields, Caroline Butler, Sarah Chappell, Srilatha Neshangi, Ingrid Camelo

**Affiliations:** Augusta University, Augusta, Georgia; Augusta University, Augusta, Georgia; Augusta University, Augusta, Georgia; Augusta University Medical Center, Augusta, Georgia; Children's Hospital of Georgia, Augusta, Georgia

## Abstract

**Background:**

A resurgence of invasive *Group A Streptococcus* (iGAS) infection in children was observed in Colorado and Minnesota by the Centers For Disease Control and Prevention (CDC) during the fall of 2022. Cases were accompanied by hospitalizations for respiratory syncytial virus (RSV) and influenza. We describe hospital admissions with iGAS and non iGAS infections at a tertiary health care center in Augusta, GA during 2021 and 2022; the only children’s Hospital with 150 licensed beds and a population of 201.196 as of 2021.

**Methods:**

We used the iGAS definition by the CDC as a Group A *Streptococcus* (GAS) isolated by culture in specimens obtained from a sterile body site, or GAS isolated from wound culture and necrotizing fasciitis or streptococcal toxic shock syndrome. We conducted a retrospective chart review of iGAS and non iGAS cases admitted to the Children’s Hospital of Georgia during January to December 2021 and January to December 2022. We collected demographics, site of infection, concomitant viral infections diagnosed by nasopharyngeal (NP) PCR, intensive care unit (ICU) admission and mortality.

**Results:**

During 2021, only 1 patient was admitted with non iGAS, a 7-year-old with a wound infection. In 2022, 15 patients were diagnosed with GAS infection, 7 of them had iGAS and 8 had noninvasive disease. We included positive peritoneal and pleural fluid, joint/bone aspirates, bronchoalveolar lavage samples, and blood cultures for iGAS. The age range for iGAS was 6-17 years and for noninvasive, 16 mo to 11 years. In 2022, 25% of noninvasive cases had concomitant influenza compared to 43% of iGAS cases. Only one patient with non iGAS tested positive for RSV. 2 patients had rhinovirus, one had adenovirus. One patient with iGAS was positive for metapneumovirus (HMPV). 57% of iGAS cases were admitted to the ICU (n=4). 50% of noninvasive cases (n=4) were admitted to the ICU. One iGAs case died. All noninvasive cases survived.

**Conclusion:**

There was increase in iGAS and noninvasive GAS infections during 2022 compared to 2021 in our institution. iGAS was more common in older children. Of iGAS cases, the only coexisting viruses were influenza and HMPV. Most cases were seen during Fall of 2022. The cause of reappearance of iGAS and non iGAS is still unknown but believed to be related to end of mask mandates for COVID 19 infections.

**Disclosures:**

**All Authors**: No reported disclosures

